# Intrahepatic activated leukocyte cell adhesion molecule induces CD6^high^CD4^+^ T cell infiltration in autoimmune hepatitis

**DOI:** 10.3389/fimmu.2022.967944

**Published:** 2022-09-09

**Authors:** Qiwei Qian, Nana Cui, Bingyuan Huang, Yudong Zhao, Qiaoyan Liu, Mingli Hu, Bo Li, Qixia Wang, Qi Miao, Zhengrui You, Xiong Ma, Ruqi Tang

**Affiliations:** ^1^ Division of Gastroenterology and Hepatology, Key Laboratory of Gastroenterology and Hepatology, Ministry of Health, State Key Laboratory for Oncogenes and Related Genes, NHC Key Laboratory of Digestive Diseases, Renji Hospital, School of Medicine, Shanghai Jiao Tong University, Shanghai Institute of Digestive Disease, Shanghai, China; ^2^ Department of Liver Surgery, Renji Hospital, School of Medicine, Shanghai Jiao Tong University, Shanghai, China

**Keywords:** autoimmune hepatitis, activated leukocyte cell adhesion molecule, CD6, migration, immune regulation

## Abstract

**Background and objectives:**

Autoimmune hepatitis (AIH) is characterized by the expansion and accumulation of pathogenic T cells in liver. Although CD6 and its ligand activated leukocyte cell adhesion molecule (ALCAM) are involved in the evolution of multiple inflammatory diseases, their roles in the pathogenesis of AIH remain unknown. Herein, we aimed to investigate ALCAM-CD6 axis in AIH development.

**Methods:**

Immunohistochemistry was performed to examine hepatic expression of CD6 and ALCAM. The concentration of serum ALCAM was evaluated by ELISA. The phenotypes of liver infiltrating T cells were determined by flow cytometry. Primary human CD4^+^ T cells were used for functional studies.

**Results:**

Our data showed that patients with AIH exhibited significantly higher expression of CD6 in the liver as compared to primary biliary cholangitis (PBC), chronic hepatitis B (CHB), non-alcoholic liver disease (NAFLD), and healthy controls (HC). In addition, hepatic CD6 expression was strongly correlated with disease severity of AIH. CD6 was mainly expressed on CD4^+^ T cells in the liver and intrahepatic CD6^high^CD4^+^ T cells demonstrated stronger proinflammatory response and proliferation features than CD6^low^ counterparts in both AIH and HC. ALCAM, the ligand of CD6, was highly expressed in the hepatocytes of AIH and serum ALCAM was strongly associated with clinical indices of AIH. Interestingly, close spatial location between CD6^+^CD4^+^ T cells and ALCAM^+^ hepatocytes was observed. Finally, we found that CD6^high^CD4^+^ T cells showed enhanced capacity of trans-endothelial migration *in vitro*, which could be promoted by recombinant ALCAM.

**Conclusions:**

Our study found that ALCAM-CD6 axis was upregulated in the AIH liver, suggesting a potential target for alleviating AIH.

## Introduction

Autoimmune hepatitis (AIH) is a chronic inflammatory liver disease characterized by an aggressive T cell-mediated response, elevated levels of immunoglobulin G (IgG), as well as the presence of autoantibodies (1). There is a female predominance with female to male ratio of 3:1 in AIH (2). The prevalence of AIH varies in different geographic regions, ranging from 4.8 to 42.9 per 100,000 population (3). The annual incidence of AIH increases robustly over the past decade from 1.37 to 2.39 per 100,000 population (4). Although standard immunosuppressive therapy significantly improves the overall survival of patients with AIH, the long-term mortality remains substantial with 10-year all-cause mortality ranging widely from 5% to 26% in different cohorts (5).

An aberrant inflammatory response is thought as the hallmark and driver of AIH (6). Perturbations of both peripheral and intrahepatic T cells have been observed in AIH development, which is characterized by defective regulatory T cells and expansion of pathogenic cytotoxic T cells (7–9). As interface hepatitis is a histological feature of AIH, pathogenic CD4^+^T cells with autoantigen specificity are enriched in this lesion (10, 11). Furthermore, there is a strong association between disease activity and abundance of liver infiltrating T cells (12). However, molecular mechanisms of activation and recruitment of T cells in AIH remain poorly elucidated.

CD6, a surface scavenger-like lymphocyte receptor, modulates the threshold for thymocyte selection and acts as a co-stimulator for peripheral T cell activation (13). Experimentally, CD6 was found to play dual roles in regulating TCR signal transmission *via* formatting different signalosomes: SLP-76/ZAP70/VAV1 for positive while UBASH3A/STS-2 for negative (14). In addition, CD6 may promote T cell trans-endothelial migration in an ARHGAP45-dependent manner (15).

Activated leukocyte cell adhesion molecule (ALCAM) and CD318 are ligands for CD6 (16). Though the role of CD318 is little known, ALCAM-CD6 signaling is essential for immune synapse stabilization and T cell proliferation (17). Increasing evidence has indicated that ALCAM is involved in multiple inflammatory diseases. For example, urinary ALCAM is a non-invasive biomarker of lupus nephritis; increased epithelial ALCAM expression supports T cell transmigration, and ALCAM expressed on B cell can navigate pathogenic B cells into the brain lesion of patients with multiple sclerosis (MS) (18–20). Blocking the ALCAM-CD6 axis, either using neutralizing antibody or genetic deletion, has shown alleviating effects for several diseases, including experimental encephalomyelitis, asthma, and even COVID-19 (21–24). Nevertheless, the role of ALCAM-CD6 axis in AIH is little known.

Here, we analyzed the histological expression of CD6 and ALCAM in the livers of AIH, and examined their correlations with clinical indices in AIH. We also compared the phenotypes between CD6^high^ and CD6^low^ subsets within intrahepatic CD4^+^ T cells isolated from AIH and HC. Moreover, the effect of ALCAM on T cell migration was investigated *in vitro*.

## Materials and methods

### Study subjects and samples

Liver biopsies at diagnosis were obtained from 61 patients with AIH, 10 with primary biliary cholangitis (PBC), 17 with chronic hepatitis B (CHB), 8 with non-alcoholic liver disease (NAFLD), and 4 healthy controls (HC). Serum was obtained from another cohort containing 86 patients with AIH and 28 HC. Liver infiltrating cells were isolated from liver explants of AIH (n=8) and healthy donors (n=9). All patients met established diagnostic criteria of AIH (25), PBC (26), NAFLD (27) and CHB (28). The clinical characteristics of the above-mentioned cohort were listed in **Supplementary Tables S1, S2, S3**, respectively. The study was approved by Ethics Committee of Renji Hospital, Shanghai Jiao Tong University.

### Immunohistochemistry and confocal staining assay

Liver biopsy samples were obtained from liver punctures, and then fixed in 10% formalin, embedded in paraffin, and cut into 4 µm sections for further immunohistochemistry and confocal assay. Immunohistochemistry staining was carried out as previously described (29). Briefly, after antigen retrieval and 3% H_2_O_2_ incubation (Beyotime, Shanghai, China, P0100A), liver sections were blocked with 10% goat serum (Solarbio, Beijing, China, SL038) for 60 minutes at room temperature and then incubated with rabbit anti-human CD6 (Abcam, Cambridge, UK, ab109217, 1:100) or rabbit anti-human ALCAM (Abcam, Cambridge, UK, ab109215, 1:50) overnight at 4°C. After washing in 1× PBS (GENOM, Haining, China, GNM20012), the sections were incubated with a horse radish peroxidase (HRP)-conjugated secondary antibody (Long Island, Shanghai, China, D-3004) at room temperature for 30 minutes and detected by 3, 3′ diaminobenzidine (MXB Biotechnologies, Fuzhou, China, MAX007) and imaged by a light microscope.

For confocal staining assay, after antigen retrieval and 3% H_2_O_2_ incubation, liver sections were blocked with 10% donkey serum (Solarbio, Beijing, China, SL050) for 60 minutes at room temperature. Then liver sections were incubated with rabbit anti-human CD6 (Abcam, Cambridge, UK, ab109217, 1:100) and mouse anti-human CD4 (eBioscience, Carlsbad, CA, USA, 14-2444-82, 1:50) or mouse anti-human CD8 (Abcam, Cambridge, UK, ab17147, 1:25) overnight at 4°C. After washing in PBS, the sections were incubated with different fluorochrome-conjugated secondary antibodies (Invitrogen, Carlsbad, CA, USA, 1:500) for 30 minutes at room temperature. Confocal scanning was performed using an LSM-710 laser-scanning confocal microscope (Carl Zeiss, Jena, Germany).

### Human liver-infiltrating cells isolation

Human liver-infiltrating cells were obtained as described previously (29). Liver resection tissues were obtained from patients with AIH who underwent liver transplantation and healthy liver donors. About 2-3×10^5^ cells per gram were isolated from the healthy liver. Due to severe liver dysfunction and failure, about 0.5-1 ×10^5^ cells per gram were isolated from the AIH liver. Briefly, excised tissues were immediately placed in RPMI 1640 medium (Gibco, Carlsbad, CA, USA, C22400500) on ice and transported to the laboratory within 30 minutes. Tissues were minced with scissors on ice and digested with 0.01% collagenase IV (Sigma-Aldrich, St. Louis, CA, USA, C4-22-1G) at 37°C for 30 minutes with agitation (220 RPM), then strained through a 70 µm cell strainer along with RPMI 1640. Then cell suspensions were gently centrifuged at 50g for 5 minutes, cell pellet was removed and supernatant was centrifuged at 750g for 10 minutes. Thereafter cell pellet was resuspended in 33% Percoll (Cytiva, Uppsala, Sweden, 17089109) and centrifuged at 900g for 30 minutes without brake. Red blood cell lysis was performed followed by removing the top fragment. Lysis procedure was stopped with RPMI 1640 containing 10% fetal bovine serum (FBS, Gibco, Carlsbad, CA, USA, 12483020) and centrifuged at 500g for 5 minutes. After removing the supernatant, cell pellet was resuspended and strained through a 30 µm cell strainer (Miltenyi Biotec, Bergisch Gladbach, Germany, 130-098-458) to remove remaining debris. Then cells were frozen for further study.

### Enzyme-linked immunosorbent assay

ALCAM levels in human serum samples were detected using the human soluble ALCAM enzyme-linked immunosorbent assay kit (Cloud-Clone Corp, Wuhan, China, SEA002Hu). The microplate provided in this kit has been pre-coated with an antibody specific to ALCAM. Human serum samples stored in the -80°C refrigerator were thawed at room temperature and diluted 10-fold by 1× PBS. Next, diluted serum or standard samples were added to microplate. Samples were removed after 1 hour of incubation. Then biotin-conjugated antibody specific to ALCAM, HRP-conjugated avidin, and 3,3’, 5,5’-tetramethylbenzidine (TMB) substrate were added and incubated sequentially. Sulphuric acid was added for terminating reaction. Finally, the color change of microplate was measured spectrophotometrically at a wavelength of 450 nm (Thermo Fisher Scientific, Vantaa, Finland, 51119200). The concentration of ALCAM in the serum was then determined by comparing the O.D. of the samples to the standard curve.

### T cell isolation, culture, and activation

CD4^+^ T cells were isolated from PBMCs of healthy donors by magnetic beads sorting with human CD4 MicroBeads (Miltenyi Biotec, Bergisch Gladbach, Germany, 130-045-101) following the manufacturer's instructions. Examined by flow cytometry, the purity of isolated CD4^+^ T cells was higher than 95% (**Supplementary Figure S1**). Then, isolated cells were cultured in complete RPMI-1640 medium supplemented with 10% heat-inactivated FBS (Gibco, Carlsbad, CA, USA, 10100147), 1% penicillin/streptomycin (Gibco, Carlsbad, CA, USA, 15140163), and 50 mM 2-mercaptoethanol (Invitrogen, Carlsbad, CA, USA, 21985023) unless otherwise described. T cell stimulation was performed as previously described (30). Briefly, cells were seeded at 5×10^5^ or 2×10^5^ cells per well in a flat-bottom 24 or 96-well plate that pre-coated with anti-CD3 (eBioscience, Carlsbad, CA, USA, 16-0037, 0.25ug/ml) for 3 days. In addition, soluble anti-CD28 (eBioscience, Carlsbad, CA, USA, 16-0289, 1ug/ml) was added. Recombinant human ALCAM Fc chimera (R&D Systems, Minneapolis, MN, USA 7187-AL, referred as rhALCAM) was added when required.

### Transwell assay

The transwell assay in this study was slightly modified from previously described methods (30–32). First, blood CD4^+^ T cells from healthy donors were isolated with human CD4 MicroBeads (Miltenyi Biotec, Bergisch Gladbach, Germany, 130-045-101) according to the manufacturer’s instructions. Isolated CD4^+^ T cells were cultured in the above-mentioned medium and incubated in a 24-well plate with 5×10_5_ cells per well. CD4^+^ T cells were stimulated with plate-bound anti-CD3 (eBioscience, Carlsbad, CA, 16-0037, 0.25ug/ml) and soluble anti-CD28 (eBioscience, Carlsbad, CA, 16-0289, 1ug/ml) for 48 hours. Next, activated CD4^+^ T cells were harvested and placed on the upper chamber of a 24-well transwell plate with 5µm pores (Corning, Kennebunk, MA, 3421) for 24 hours. To investigate whether ALCAM induces trans-endothelial migration of T cells, rhALCAM (3ug/ml) was added to the lower chamber, with vehicle (1× PBS) as control. Then cells in lower chamber were counted with hemacytometers.

### Cell proliferation assay

Magnetically selected CD4^+^ T cells were labeled with 2.5μM CellTrace carboxyfluorescein succinimidyl ester (CFSE) (Invitrogen, Carlsbad, CA, USA, C34554) in PBS containing 5% FBS as described (33). Then labeled CD4^+^ T cells were activated with pre-coated anti-CD3 and soluble anti-CD28 for 72 hours, the proliferation of T cells was assessed by flow cytometry.

### Flow cytometry

For intracellular cytokines detection, cells were incubated in complete RPMI-1640 containing 10% FBS and leukocytes activation cocktail with GolgiPlug (BD Biosciences, San Diego, CA, USA, 550583) in a 37°C humidified CO_2_ incubator for 5 hours. Next, cells were stained with live/dead, and surface markers, fixed with the Fix/Perm kit (BD Biosciences, San Diego, CA, USA, 554714) and incubated with antibodies against intracellular cytokines. For detecting transcription factors, cells were first stained with live/dead and cell surface markers. After fixed and washed with the transcription factor buffer set (BD Biosciences, San Diego, CA, USA, 562574), cells were stained for nuclear factors as manual. Monoclonal antibodies specific to CD3 (HIT3a), CD4 (SK3), CD8 (SK1), CD69 (FN50), CD103 (Ber-ACT8), LAG-3 (T47-530), T-bet (O4-46) were purchased from BD Biosciences (San Diego, CA, USA); CD6 (BL-CD6), PD-1 (EH12.2H7), IFN-γ (4S.B3), TNF-α (MAb11), IL-2 (MQ1-17H12) and IL-17A (BL168) were purchased from Biolegend (San Diego, CA, USA); CD3 (OKT3), EOMES (WD1928), TOX (TXRX10) and Ki-67 (SolA15) were purchased from eBioscience (eBioscience, Carlsbad, CA, USA). All samples were detected by flow cytometry (Celesta, BD Bioscience) and analyzed using FlowJo software (10.6.2, Tree Star).

All reagents and related applications involved in this study were listed in **Supplementary Table S4**.

### Statistical analysis

All statistical analyses were performed with GraphPad Prism 8.3. Statistical differences for normally distributed data were analyzed by Student’s t-test. Correlations were analyzed using correlation coefficient. All analyses were two-tailed, and p < 0.05 was considered significant.

## Results

### CD6 expression was increased in AIH liver and correlated with disease severity

**Figure 1:**
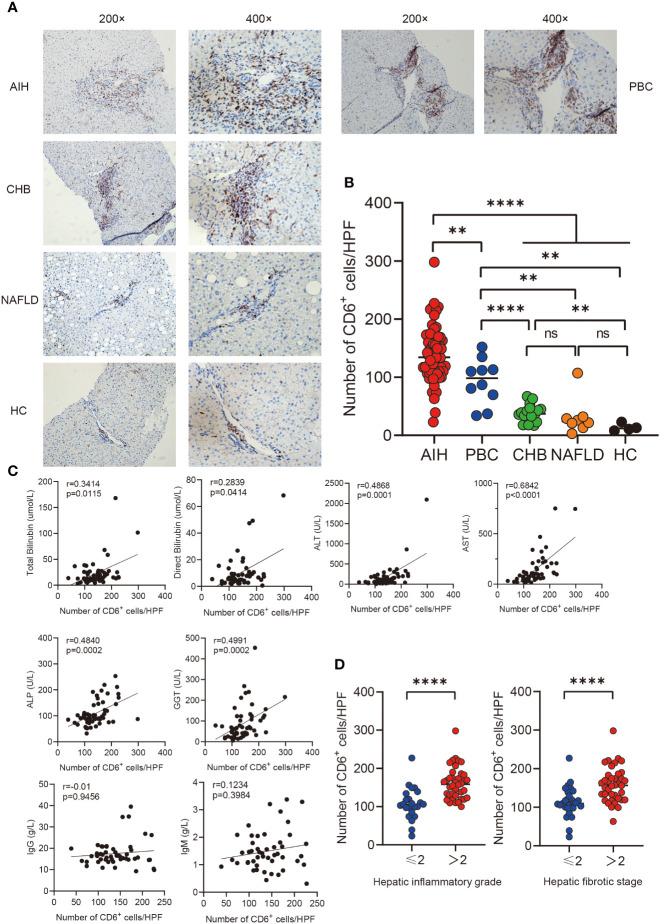
Immunohistochemistry analysis of CD6 expression in liver**. (A, B)** Representative immunohistochemistry images and quantification of CD6 positive cells in liver biopsies from AIH (n=61), PBC (n=10), CHB (n=17), NAFLD (n=8) and HC (n=4). **(C)** Correlations between the number of CD6 positive cells and clinical features of patients with AIH (n=61). **(D)** The number of CD6 positive cells was compared between different inflammatory grades and fibrosis stages. **p < 0.01, ****p < 0.0001, ns: not significant.

In order to investigate the expression of CD6 in various inflammatory liver diseases, immunohistochemistry was performed on liver biopsies from individuals with autoimmune hepatitis (AIH, n=61), primary biliary cholangitis (PBC, n=10), non-alcoholic liver disease (NAFLD, n=8), chronic hepatitis B (CHB, n=17) and healthy controls (n=4). CD6 expression was significantly higher in the AIH liver as compared to PBC (p < 0.01), NAFLD, CHB and HC (p < 0.0001, **Figures 1A, B**). Next, we assessed the correlation between CD6 expression and clinical indicators of patients with AIH. Interestingly, the number of CD6^+^ cells was positively correlated with multiple serological markers, including total bilirubin (TB, r = 0.3414, p = 0.0115), direct bilirubin (DB, r = 0.2839, p = 0.0414), alanine transaminase (ALT, r = 0.4868, p = 0.0001), aspartate transaminase (AST, r = 0.6842, p < 0.0001), alkaline phosphatase (ALP, r = 0.4840, p = 0.0002), and gamma-glutamyl transferase (GGT, r = 0.4991, p = 0.0002). However, no significant correlation was found between CD6^+^ cells and IgG or immunoglobulin M (IgM) (**Figure 1C**). We further classified the patients with respect to their inflammation degrees and fibrosis stages. As shown in **Figure 1D**, the number of CD6^+^ cells was significantly higher in patients with advanced inflammation and fibrosis stages. In sum, these data suggest that CD6 is involved in AIH inflammation and may track with disease severity.

### CD6 was mainly expressed on CD4^+^ T cells in the liver of AIH

**Figure 2:**
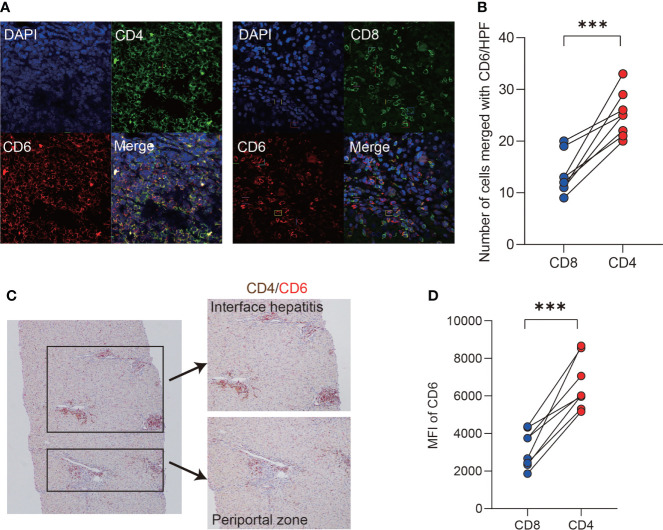
CD6 was mainly expressed on CD4^+^ T cells in AIH liver. **(A, B)** Representative confocal images and quantification analysis of CD4^+^CD6^+^, CD8^+^CD6^+^ cells using human liver biopsies from patients with AIH (n=8). **(C)** Representative dual-color immunohistochemistry images of CD4 (brown) and CD6 (red) using human liver biopsies from patients with AIH (n=10). **(D)** The expression of CD6 on CD4^+^ and CD8^+^ T cells of liver-infiltrating cells from explanted liver of AIH was analyzed by flow cytometry (n=8). MFI, mean fluorescent intensity, ***p < 0.001.

Probing the expression of CD6 across 18 kinds of resting immune cells (34) revealed that CD6 was highly expressed on T lymphocytes. Therefore, we further investigated the expression of CD6 on T cell subsets in the AIH liver by immunofluorescence confocal staining. As shown, both CD4 and CD8 co-localized with CD6 in inflammatory niche of AIH (**Figure 2A**). Notably, the number of CD4^+^CD6^+^ T cells was higher than CD8^+^CD6^+^ counterparts (**Figure 2B**). Then double immunohistochemical staining was performed to determine the spatial distribution of CD4^+^CD6^+^ T cells in the liver of AIH. As shown in **Figure 2C**, these cells were primarily accumulated in the lesions of interface hepatitis and portal area. To confirm the data obtained by histological and transcriptomic studies, we further examined the expression of CD6 on isolated liver infiltrating cells. Flow cytometry validated significantly higher CD6 expression on CD4^+^ T cells compared to the CD8^+^ compartment in AIH (**Figure 2D**).

### Liver-infiltrating CD6^high^CD4^+^ T cells demonstrated proinflammatory features

**Figure 3:**
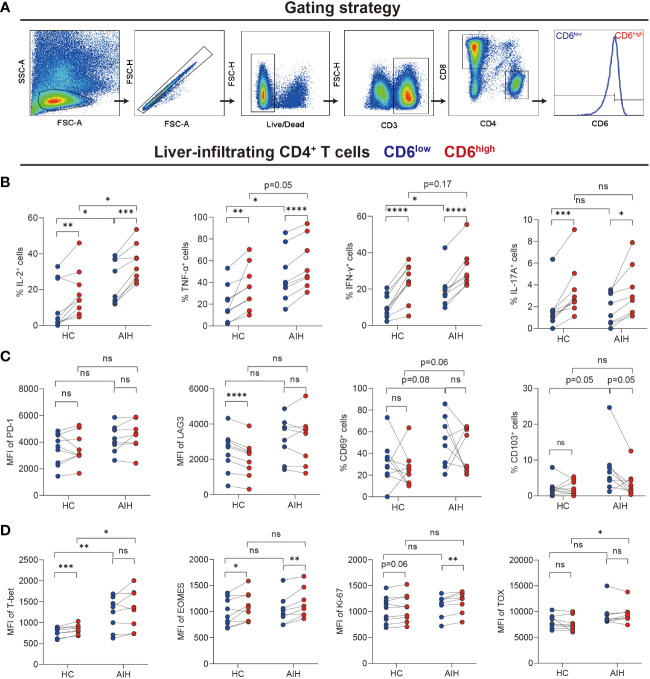
Intrahepatic CD6^high^CD4^+^ T cells demonstrated proinflammatory functions. Paired CD6^high^ and CD6^low^ cells among CD4^+^ T cells of liver-infiltrating cells from healthy donors (n=9) and patients with AIH (n=8) were analyzed by flow cytometry. **(A)** The gating strategy for grouping intrahepatic CD4^+^ T cells into CD6^high^ and CD6^low^ subsets. **(B–D)** The expression of intracellular cytokines, surface markers, and transcription factors on CD6^high^ and CD6^low^ subsets from the liver of HC (n=9) and AIH (n=8). *p < 0.05, **p < 0.01, ***p < 0.001, ****p < 0.0001, ns: not significant.

We next investigated the immunological features of intrahepatic CD4^+^CD6^+^ T cells by flow cytometry. CD4^+^ T cells were grouped into CD6^high^ and CD6^low^ subsets manually (**Figure 3A**). First, cytokines, cell surface markers and transcription factors were compared between the CD6^high^ and CD6^low^ subsets in AIH and HC, respectively (pathological and physiological roles of these markers were summarized in **Supplementary Table S5**). We observed that CD6^high^CD4^+^ T cells expressed more proinflammatory cytokines than CD6^low^CD4^+^ T cells, including TNF-α, IFN-γ, IL-2 and IL-17A in both HC and AIH (**Figure 3B**). No significant difference of co-inhibitory or tissue-resident markers was observed between these two subsets in either HC or AIH, except that CD6^high^ subset produced lower level of lymphocyte activation gene-3 (LAG-3) than CD6^low^ subset in HC (**Figure 3C**). Furthermore, we examined transcription factors associated with activation, exhaustion and proliferation. The CD6^high^ subset expressed higher T-box-expressed-in-T-cells (T-bet) and eomesodermin (EOMES) than the CD6^low^ subset in HC while upregulated EOMES and marker of proliferation Ki-67 were observed in the CD6^high^ subset in AIH. When comparing CD6^high^CD4^+^ T cells between AIH and HC groups, this cell population from AIH produced significantly more IL-2 and TNF-α, as well as T-bet and thymocyte selection-associated high mobility group box protein (TOX) than those from HC (**Figures 3B, D**). Taken together, these data implicate that liver-infiltrating CD6^high^CD4^+^ T cells exhibited proinflammatory functions and showed altered phenotypes in patients with AIH.

### The expression of ALCAM was significantly elevated in AIH

**Figure 4:**
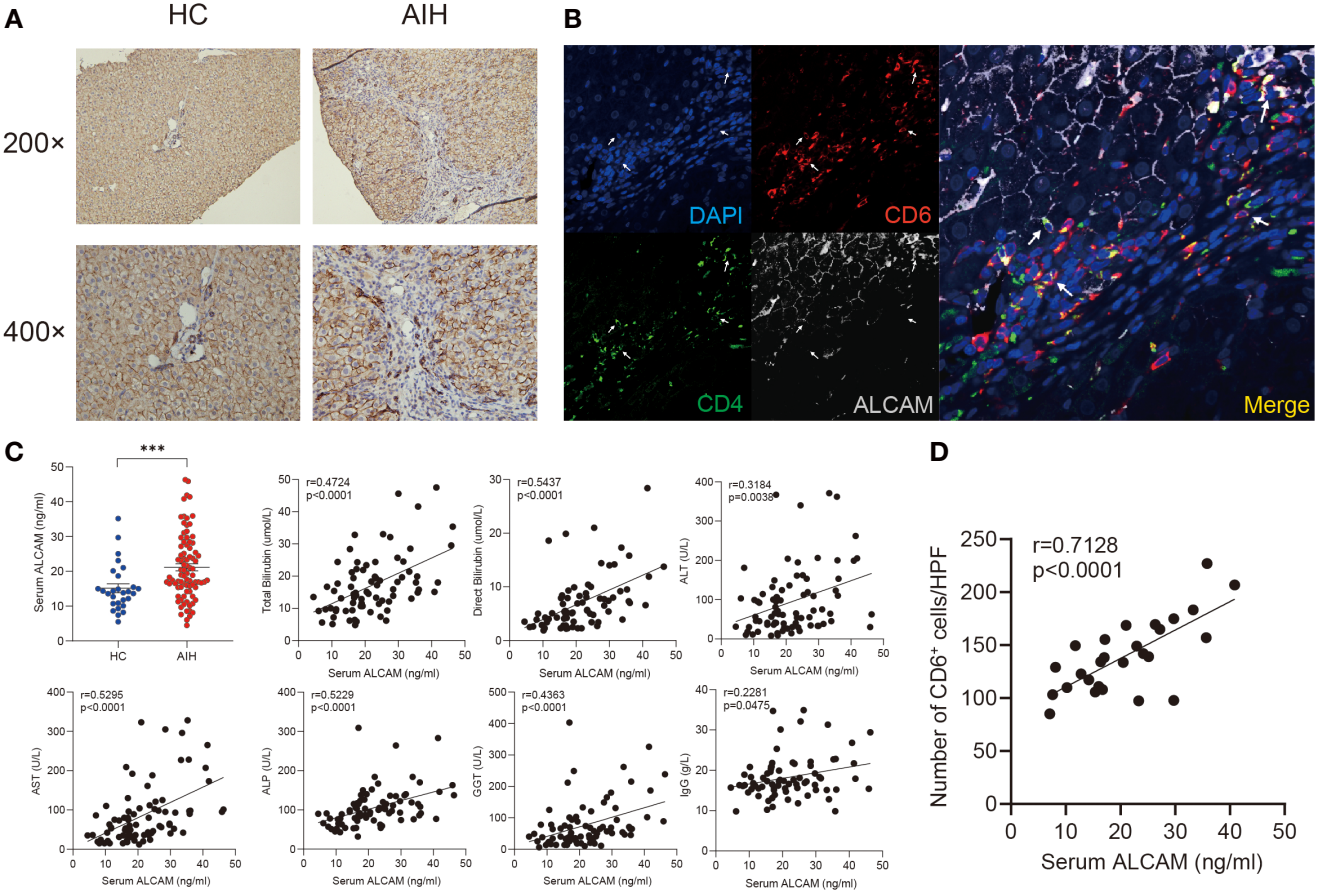
Elevated hepatic and serum ALCAM were observed in patients with AIH. **(A)** Representative immunohistochemistry images of ALCAM in liver biopsies from HC (n=3) and patients with AIH (n=6). **(B)** Representative immunofluorescence staining for CD4, CD6 and ALCAM in interface hepatitis lesion of liver sections from patients with AIH (n=3). **(C)** Concentration of serum ALCAM in HC (n=28) and AIH (n=86) was measured by ELISA assay. Individual correlation between clinical indicators and serum ALCAM was calculated in patients with AIH (n=86). **(D)** Correlation between the number of hepatic CD6^+^ cells and paired serum ALCAM concentration was calculated (n=27). ***p < 0.001.

ALCAM have been identified as a canonical ligand for CD6 (35). Immunohistochemical staining showed ALCAM was predominantly expressed on the hepatocytes surrounded by infiltrated immune cells in the AIH liver compared with HC (**Figure 4A**). More importantly, we observed the close location between CD6^+^CD4^+^ T cells and ALCAM^+^ hepatocytes in the AIH liver (**Figure 4B**).

Previous studies have revealed that membranous ALCAM could be cut as a soluble form and used for tracking disease activity in multiple inflammatory diseases (36). Thus, serum ALCAM levels were measured in a cohort including 86 patients with AIH and 28 healthy controls. Serum ALCAM levels were significantly higher in AIH compared to healthy controls (21.4 ± 1.0 vs 14.4 ± 1.2ng/ml, p < 0.0001). Furthermore, serum ALCAM levels were strongly correlated with liver injury parameters, including total bilirubin (r = 0.4724, p < 0.0001), direct bilirubin (r = 0.5437, p < 0.0001), ALT (r = 0.3184, p = 0.0038), AST (r = 0.5295, p<0.0001), ALP (r = 0.5229, p < 0.0001) and GGT (r = 0.4363, p = 0.0003) while it demonstrated mild correlation with IgG (r = 0.2281, p = 0.0475) (**Figure 4C**).

In addition, paired serum samples from 27 patients with AIH at the time of liver biopsy were obtained. We further investigated the potential relationship between serum ALCAM and hepatic CD6^+^ cells. Intriguingly, a significant correlation between serum ALCAM concentration and the number of hepatic CD6^+^ cells was observed (r=0.7128, p<0.0001, **Figure 4D**). Taken together, these data suggest a potential role of ALCAM-CD6 axis in the development of AIH.

### ALCAM promoted CD6^high^CD4^+^ T cells migration *in vitro*

**Figure 5:**
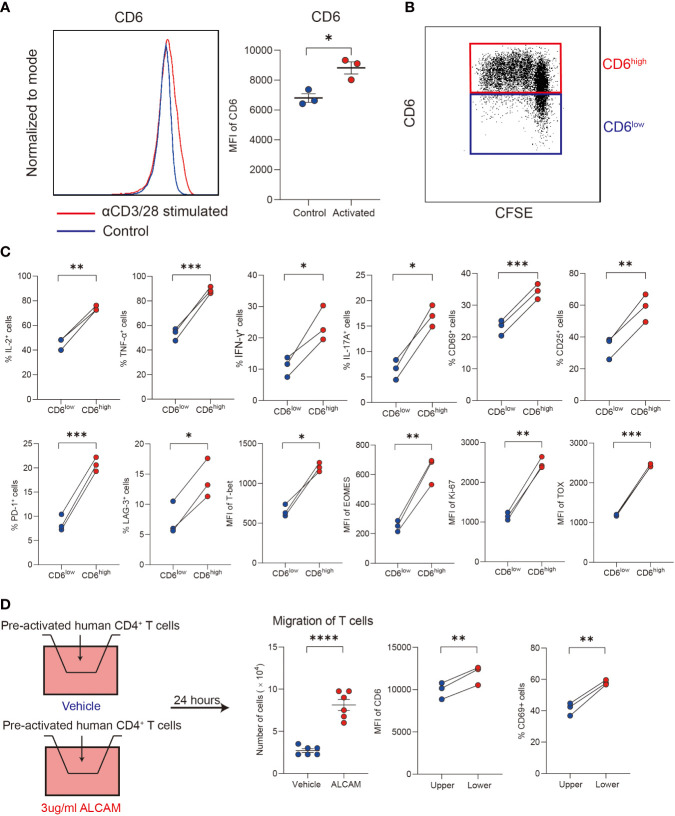
ALCAM promoted CD6^high^CD4^+^ T cells trans-endothelial migration *in vitro*. **(A, B)** Human CD4^+^ T cells were magnetically isolated from PBMC of healthy donors and stimulated with αCD3/28 for 3 days in a flat-bottom 96-well plate, the expression of CD6 and cell proliferation was detected with flow cytometry. **(C)** After stimulation, the expression of cytokines, surface markers and transcription factors was compared between the CD6^high^ and CD6^low^ subsets. **(D)** Pre-activated CD4^+^ T cells were placed on a transwell chamber with 5μm pore for 24 hours in the presence of rhALCAM (3ug/ml) or vehicle (PBS). The expression of CD6 and CD69 was measured by flow cytometry. Experiments were repeated at least three times. *p < 0.05, **p < 0.01, ***p < 0.001, ****p < 0.0001.

Primary human CD4^+^ T cells isolated from PBMCs of healthy donors were used to explore the immunological functions of CD6^high^CD4^+^ T cells *in vitro*. After stimulated with αCD3/28 for 3 days, a significant upregulation of CD6 was observed (**Figure 5A**). Compared with the CD6^low^ subset, the CD6^high^ subset demonstrated robust proliferation capacity (**Figure 5B**) and produced more IL-2, TNF-α, IFN-γ, IL-17A and expressed higher CD25 and CD69, which implicated its proinflammatory and activated status *in vitro* (**Figure 5C**).

We next investigated the migration ability of different T cell subsets. Primary CD4^+^ T cells were pre-activated for 48 hours, and then cells were placed in the transwell chamber for 24 hours. Given the increased expression of ALCAM and infiltration of CD6^high^CD4^+^ T cells in AIH liver, we hypothesized that ALCAM may predispose the invasion of CD6^high^CD4^+^ T cells. As a result, a significantly higher number of cells in the low chamber was observed after adding rhALCAM in culture medium, indicating the potential role of ALCAM in promoting CD6^high^CD4^+^ T cells migration. Moreover, we found that T cells in the lower chamber expressed higher CD6 and CD69 than the compartments in the upper chamber, suggesting the enhanced migration ability of CD6^high^ T cells (**Figure 5D**). In summary, these data suggest that ALCAM may promote the infiltration of CD6^high^CD4^+^ T cells in the liver of AIH.

## Discussion

The accumulation of liver-damaging CD4^+^ T cells and impairment of regulatory CD4^+^ subsets are thought to contribute to the development of AIH (9). Compared to HC, we first identify increased hepatic expression of CD6 and serum ALCAM in AIH, which was positively correlated with disease severity. In contrast to the CD6^low^ subset, CD6highCD4^+^ T cells are more pathogenic characterized by producing more proinflammatory cytokines such as TNF-α, IFN-γ, IL-2, IL-17A, as well as exhibiting increased proliferative ability. Furthermore, CD6^high^CD4^+^ T cells in AIH show even higher expression of TNF-α, and IL-2 than those cells in HC. Notably, we identify that the CD6^high^ subset shows a more robust function of trans-endothelial migration than the CD6^low^ subset.

CD6 acts as a hub that regulates both stimulatory and inhibitory signaling after TCR activation. After phosphorylation of intracellular structures, CD6 can bind to multiple proteins for assembling various signalosomes. Previous interaction proteomics study reveals that CD6 aggregates with SLP-76, ZAP70 and VAV-1 to transmit T cell activation signaling, and the interaction with UBASH3A/STS-2 is an inhibitor for negative regulation. Growing evidence has emphasized the clinical significance of CD6 in immune-mediated disorders (14). Genetically, single nucleotide polymorphisms of CD6 have been reported in the evolution of multiple autoimmune diseases, such as MS, rheumatoid arthritis and Behcet’s disease (37, 38). The lesion-infiltrating CD6^high^CD4^+^ population also demonstrates pathogenic phenotypes in patients with IBD and MS (30, 39). In accordance with these studies, we observed the increased abundance of aggressive CD6^high^CD4^+^ T cells in AIH liver, which may be responsible for the liver damage.

Liver microenvironment plays a crucial role in establishing and maintaining phenotypes of infiltrating CD4^+^ cells (40). Under pathological conditions, injured hepatocytes provide an environment for cytotoxic cells differentiation and proliferation. Generally, excessive migration and infiltration of immune cells into local tissue is the key feature of inflammation. This procedure depends primarily on specific chemokines and adhesion molecules. In blood-brain barrier, Cayrol et al. points out that upregulated ALCAM, which is known as a canonical ligand for CD6, replaces other adhesion molecules such as ICAM-1 or VCAM-1 for lymphocytes transmigration (19). Herein, we observe elevated hepatic ALCAM in AIH. The spatial proximity between CD6^+^CD4^+^ T cells and ALCAM^+^ hepatocytes is observed in the interface hepatitis lesion. Although we hardly observe the functional effect of ALCAM on human CD4^+^ T cells such as cytokine-production and activation, ALCAM could promote trans-endothelial migration of CD6^high^CD4^+^ T cells. Therefore, increased ALCAM may be involved in the evolution of hepatitis by driving the hepatic enrichment and infiltration of CD6^high^CD4^+^ T cells in AIH.

Moreover, multiple studies have underlined the significance of ALCAM-CD6 interaction in inflammatory diseases. Interestingly, significant evidence suggests that ALCAM participates in the initiation of inflammatory responses regardless of the subtypes of inflammation, such as Th1/17 mediated inflammatory bowel diseases, multiple sclerosis and Th2 mediated asthma or atopic dermatitis (21, 22, 30, 41), which implicates the conserved role of ALCAM for enriching pathogenic T cells in the lesions. Recently, monoclonal antibody Itolizumab, which targets the ALCAM-CD6 interaction, has been proved to alleviate several inflammatory conditions, such as arthritis, psoriasis and Covid-19 (42–44). Itolizumab robustly suppresses the production of proinflammatory cytokines and proliferation of Th1/17 cells (45). However, both *in vitro* and clinical studies have revealed that the anti-inflammatory effect of Itolizumab on CD4^+^ T cells is lost at the highest doses (46). Therefore, it is a promising intervention for AIH treatment by blocking the ALCAM-CD6 signaling, which still needs more trials to determine the appropriate dose and procedure.

**Figure 6:**
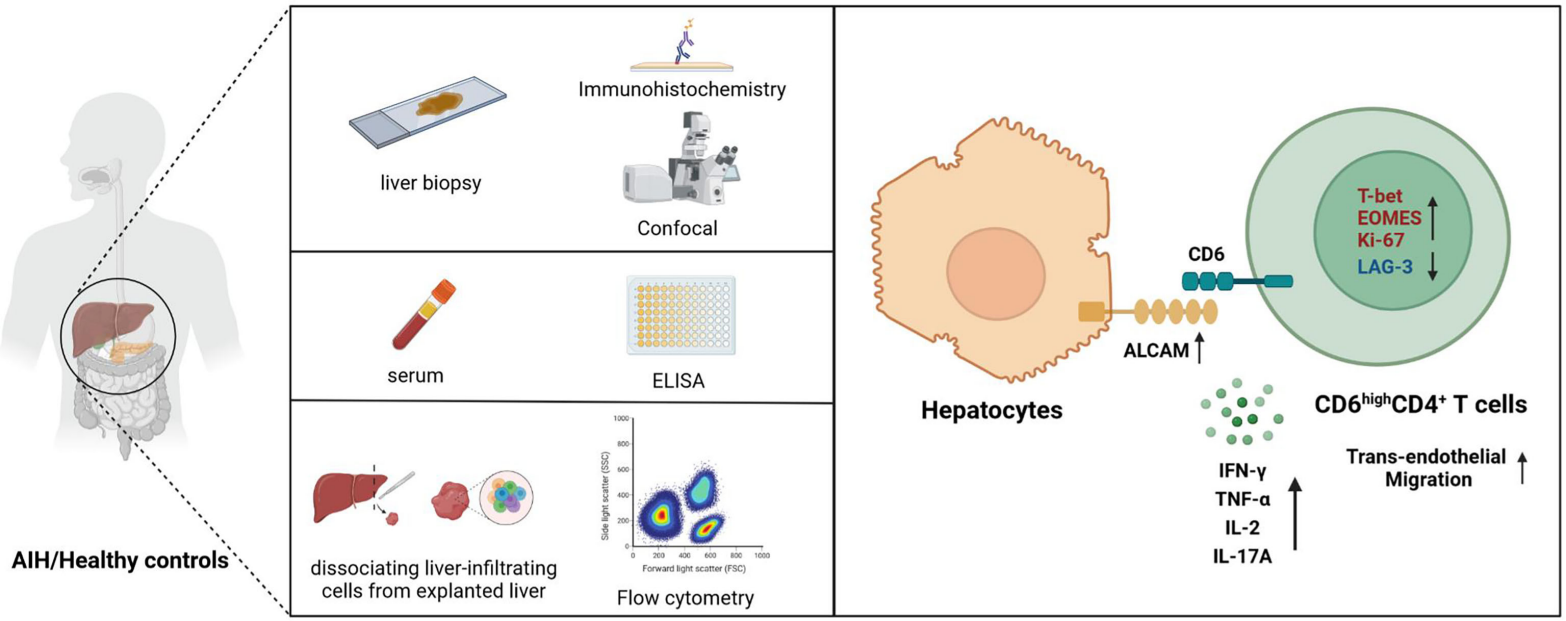
Schematic diagram for this study. Upregulated ALCAM on hepatocytes promoted the trans-endothelial migration of pathogenic CD6^high^CD4^+^ T cells, which further aggravated hepatic inflammation of patients with AIH. This study revealed a putative therapeutic approach for patients with AIH.

In conclusion, our findings suggest that overexpressed hepatic ALCAM may be involved in AIH pathogenesis by recruiting proinflammatory CD6^high^CD4^+^ T cells (**Figure 6**). Combining ALCAM-CD6 blockade with existing management assays may be a feasible approach to alleviate AIH.

## Data availability statement

The original contributions presented in the study are included in the article/**Supplementary Material**. Further inquiries can be directed to the corresponding authors.

## Ethics statement

The studies involving human participants were reviewed and approved by Ethics Committee of Renji Hospital, Shanghai Jiao Tong University. The patients/participants provided their written informed consent to participate in this study.

## Author contributions

RT, ZY and XM designed the study. QQ and NC performed the experiments. QQ, NC, BH, YZ, QL, MH, BL, QW and QM collected the samples. QQ and NC analyzed the data. QQ and NC wrote the manuscript. ZY, RT and XM revised the manuscript. RT, XM and QW obtained the funding. All authors contributed to the article and approved the submitted version.

## Funding

This work was supported by the National Natural Science Foundation of China grants (#81922010, and 81873561 to RT; #81830016, 81771732, and 8213000085 to XM; #82070581 to QW) and Shanghai Municipal Education Commission and Shanghai Education Development Foundation (No. 20XD1422500 to RT).

## Acknowledgments

We appreciate all the subjects who provided samples in the study.

## Conflict of interest

The authors declare that the research was conducted in the absence of any commercial or financial relationships that could be construed as a potential conflict of interest.

## Publisher’s note

All claims expressed in this article are solely those of the authors and do not necessarily represent those of their affiliated organizations, or those of the publisher, the editors and the reviewers. Any product that may be evaluated in this article, or claim that may be made by its manufacturer, is not guaranteed or endorsed by the publisher.
