# Supplementary material for: Intrahepatic activated leukocyte cell adhesion molecule induces CD6highCD4+ T cell infiltration in autoimmune hepatitis
Source: Front Immunol. 2022 Sep 9;13:967944. doi: 10.3389/fimmu.2022.967944 (PMC9500242; doi:10.3389/fimmu.2022.967944)
Supplement: Supplementary file 1 [file DataSheet_1.docx]

Supplementary Material

# Supplementary Tables

**Table S1. Demographic and clinical features of individuals involved in IHC study.**

|  | **AIH (n=61)** | **PBC (n=10)** | **NAFLD (n=8)** | **CHB (n=17)** | **HC (n=4)** |
| --- | --- | --- | --- | --- | --- |
| **Age (years)** | 48.6±1.7 | 40.9±3.5 | 37.0±3.3 | 36.7±2.4 | 34.5±5.6 |
| **Gender (F/M)** | 51/10 | 10/0 | 5/3 | 6/11 | 1/3 |
| **Total Bilirubin (umol/L)** | 23.0±3.6 | 12.4±0.9 | 14.3±3.3 | 15.4±1.7 | 9.9±0.6 |
| **Direct Bilirubin (umol/L)** | 10.1±1.7 | 4.7±0.6 | 4.6±1.3 | 5.4±1.2 | 3.8±0.3 |
| **ALT (U/L)** | 176.4±40.0 | 89.7±20.4 | 61.5±16.7 | 30.2±4.6 | 25.3±2.4 |
| **AST (U/L)** | 137.3±20.8 | 51.2±10.2 | 35.9±6.3 | 24.5±1.7 | 24.3±0.7 |
| **ALP (U/L)** | 109.8±6.5 | 118.3±26.8 | 90.6±11.7 | 71.9±4.4 | 84.3±14.9 |
| **GGT (U/L)** | 87.0±11.2 | 112.4±25.6 | 53.2±19.9 | 22.1±4.6 | 17.1±2.9 |
| **IgG (g/L)** | 17.6±0.9 | 17.4±2.7 | 14.6±0.8 | NA | NA |
| **IgM (g/L)** | 1.5±0.1 | 2.3±0.5 | 1.4±0.2 | NA | NA |

Note: Continuous data are shown as mean ± standard error (SEM).

Abbreviations: F/M, female/male; NA, not applicable.

**Table S2. Demographic and clinical features of individuals involved in ELISA study.**

|  | **AIH (n=86)** | **HC (n=28)** |
| --- | --- | --- |
| **Age (years)** | 50.3±1.3 | 40.6±2.6 |
| **Gender (F/M)** | 75/11 | 19/9 |
| **Total Bilirubin (umol/L)** | 17.1±1.0 | 11.3±0.8 |
| **Direct Bilirubin (umol/L)** | 7.1±0.6 | 3.9±0.2 |
| **ALT (U/L)** | 95.0±9.3 | 13.0±1.1 |
| **AST (U/L)** | 87.3±8.5 | 15.6±0.9 |
| **ALP (U/L)** | 105.8±5.4 | 60.1±3.6 |
| **GGT (U/L)** | 76.9±8.4 | 16.8±1.2 |
| **IgG (g/L)** | 18.2±0.6 | NA |
| **IgM (g/L)** | 1.5±0.1 | NA |

Note: Continuous data are shown as mean ± standard error (SEM).

Abbreviations: F/M, female/male; NA, not applicable.

**Table S3. Demographic and clinical features of individuals involved for isolating liver-infiltrating cells.**

|  | **AIH (n=8)** | **HC (n=9)** |
| --- | --- | --- |
| **Age (years)** | 51.4±4.5 | 27.6±1.6 |
| **Gender (F/M)** | 7/1 | 8/1 |
| **Total Bilirubin (umol/L)** | 306.4±88.4 | 10.7±1.2 |
| **Direct Bilirubin (umol/L)** | 203.6±62.7 | 3.2±0.4 |
| **ALT (U/L)** | 183.9±86.0 | 18.1±3.1 |
| **AST (U/L)** | 162.3±94.1 | 19.0±1.5 |
| **ALP (U/L)** | 105.9±19.3 | 100.4±8.2 |
| **GGT (U/L)** | 38.8±11.7 | 19.4±3.9 |
| **IgG (g/L)** | 17.9±0.1 | NA |
| **IgM (g/L)** | 2.2±0.5 | NA |

Note: Continuous data are shown as mean ± standard error (SEM).

Abbreviations: F/M, female/male; NA, not applicable.

**Table S4. Reagents, instruments, and related application involved in this study.**

| **Reagent or Resource** | **Source** | **Identifier** | **Usage** |
| --- | --- | --- | --- |
| **Antibodies** | | | |
| Fixable Viability Stain 780 | BD Biosciences | Cat# 565388 | Flowcytometry, 1:1000 |
| anti-human CD3, BV510, Clone HIT3a | BD Biosciences | Cat# 564713 | Flowcytometry, 1:100 |
| anti-human CD4, BB700, Clone SK3 | BD Biosciences | Cat# 566392 | Flowcytometry, 1:100 |
| anti-human CD4, BV786, Clone SK3 | BD Biosciences | Cat# 563877 | Flowcytometry, 1:100 |
| anti-human CD8, BV605, Clone SK1 | BD Biosciences | Cat# 564116 | Flowcytometry, 1:100 |
| anti-human CD69, APC-R700, Clone FN50 | BD Biosciences | Cat# 565154 | Flowcytometry, 1:100 |
| anti-human CD103, BB515, Clone Ber-ACT8 | BD Biosciences | Cat# 564578 | Flowcytometry, 1:100 |
| anti-human LAG-3, APC-R700, Clone T47-530 | BD Biosciences | Cat# 565774 | Flowcytometry, 1:100 |
| anti-human T-bet, BV786, Clone O4-46 | BD Biosciences | Cat# 564141 | Flowcytometry, 1:50 |
| anti-human CD6, PE, Clone BL-CD6 | Biolegend | Cat# 313906 | Flowcytometry, 1:100 |
| anti-human PD-1, BV421, Clone EH12.2H7 | Biolegend | Cat# 329920 | Flowcytometry, 1:100 |
| anti-human IL-2, APC, Clone MQ1-17H12 | Biolegend | Cat# 500310 | Flowcytometry, 1:50 |
| anti-human IFN-γ, FITC, Clone 4S.B3 | Biolegend | Cat# 502506 | Flowcytometry, 1:50 |
| anti-human TNF-α, BV510, Clone MAb11 | Biolegend | Cat# 502950 | Flowcytometry, 1:50 |
| anti-human IL-17A, BV421, Clone BL168 | Biolegend | Cat# 512322 | Flowcytometry, 1:50 |
| anti-human CD3, Percp-Cy5.5, Clone OKT3 | eBioscience | Cat# 45-0037-42 | Flowcytometry, 1:100 |
| anti-human EOMES, PE-Cy7, Clone WD1928 | eBioscience | Cat# 25-4877-42 | Flowcytometry, 1:50 |
| anti-human/mouse TOX, eFluor 660, Clone TXRX10 | eBioscience | Cat# 50-6502-82 | Flowcytometry, 1:50 |
| anti-human Ki-67, FITC, Clone SolA15 | eBioscience | Cat# 11-5698-82 | Flowcytometry, 1:50 |
| Rabbit anti-human CD6 | abcam | Cat# ab109217 | Immunohistochemistry/Confocal, 1:100 |
| Mouse anti-human CD4 | eBioscience | Cat# 14-2444-82 | Immunohistochemistry/Confocal, 1:50 |
| Mouse anti-human CD8 | abcam | Cat# ab17147 | Immunohistochemistry/Confocal, 1:25 |
| Rabbit anti-human ALCAM | abcam | Cat# ab109215 | Immunohistochemistry/Confocal, 1:50 |
| Donkey anti-Mouse IgG (H+L) Highly Cross-Adsorbed Secondary Antibody, Alexa Fluor 488 | Invitrogen | Cat# A-21202 | Confocal, 1:500 |
| Donkey anti-Rabbit IgG (H+L) Highly Cross-Adsorbed Secondary Antibody, Alexa Fluor™ 555 | Invitrogen | Cat# A-31572 | Confocal, 1:500 |
| Mouse anti-human CD3, Functional Grade, Clone OKT3 | eBioscience | Cat# 16-0037 | Cell culture, 0.25ug/ml |
| Mouse anti-human CD28, Functional Grade, Clone CD28.2 | eBioscience | Cat# 16-0289 | Cell culture, 1ug/ml |
| Goat anti-mouse/rabbit HRP-conjugated secondary antibody | Long Island antibody | Cat# D-3004 | Immunohistochemistry, 50ug/ml |
| **Chemicals, Kits and Recombinant Proteins** | | | |
| Goat Serum | Solarbio | Cat# SL038 | Immunohistochemistry/Confocal, 10% |
| Donkey Serum | Solarbio | Cat# SL050 | Immunohistochemistry/Confocal, 10% |
| DAB Detection Kit | MXB Biotechnologies | Cat# MAX007 | Immunohistochemistry |
| EDTA | MXB Biotechnologies | Cat# MVS-0099 | Immunohistochemistry/Confocal, pH=9.0 |
| Red blood cell lysis buffer | Sigma-Aldrich | Cat# R7757 | 3ml per sample |
| Recombinant human ALCAM Fc chimera | R&D Systems | Cat# 7187-AL | Cell culture |
| ELISA Kit for Activated Leukocyte Cell Adhesion Molecule (ALCAM), human | Cloud-Clone Corp | Cat# SEA002Hu | ELISA |
| Dual-color immunohistochemistry kit | ZSGB-Bio | Cat# DS-0003 | Immunohistochemistry |
| 3% H2O2 | Beyotime | Cat# P0100A | Immunohistochemistry/Confocal |
| Ficoll Paque Plus | Cytiva | Cat# 17144003 | NA |
| Percoll | Cytiva | Cat# 17089109 | NA |
| RPMI 1640 medium | Gibco | Cat# C22400500 | Cell culture |
| Fetal bovine serum | Gibco | Cat# 12483020 | Cell culture, 10% |
| Fetal bovine serum, heat-inactivated | Gibco | Cat# 10100147 | Cell culture, 10% |
| Penicillin-Streptomycin | Gibco | Cat# 15140163 | Cell culture, 100U/ml |
| 2-mercaptoethanol | Gibco | Cat# 21985023 | Cell culture, 50mM |
| Collagenase IV | Sigma-Aldrich | Cat# C4-22-1G | Tissue digestion, 0.01% |
| MACS SmartStrainers (30 µm) | Miltenyi Biotec | Cat# 130-098-458 | NA |
| CD4 MicroBeads, human | Miltenyi Biotec | Cat# 130-045-101 | NA |
| LS Columns | Miltenyi Biotec | Cat# 130-042-401 | NA |
| LS Column Adapter | Miltenyi Biotec | Cat# 130-090-544 | NA |
| MACS BSA Stock Solution | Miltenyi Biotec | Cat# 130-091-376 | NA |
| AutoMACS Rinsing Solution | Miltenyi Biotec | Cat# 130-091-222 | NA |
| Transwell Permeable Supports, 5um, 24wells | Corning | Cat# 3421 | NA |
| 1× Phosphate buffered saline | GENOM | Cat# GNM20012 | 0.01mmol/L |
| CellTrace carboxyfluorescein succinimidyl ester (CFSE) | Invitrogen | Cat# C34554 | NA |
| Leukocytes activation cocktail, with GolgiPlug | BD Biosciences | Cat# 550583 | Flowcytometry, 1:500 |
| Cytofix/Cytoperm™ Fixation/Permeablization Kit | BD Biosciences | Cat# 554714 | Intracellular cytokine analysis |
| Transcription Factor Buffer Set | BD Biosciences | Cat# 562574 | Intracellular transcription factor analysis |
| **Instruments** | | | |
| BD FACSCelesta Multicolor Flow Cytometer | BD Biosciences | NA | Laser: Blue 488nm, Violet 405nm, Red 640nm, Yellow/green 561nm  Software: BD-FACSDiva (v9.0) |
| LSM-710 laser-scanning confocal microscope | Carl Zeiss | NA | Laser: 405\488\561\639nm |
| Multiskan GO Microplate Spectrophotometer | Thermo Fisher Scientific | Cat# 51119200 | ELISA, Laser: 450nm |

Table S5. Summary of pathological and physiological roles of the cytokines, surface markers, and transcription factors related to Figure 3.

|  | Physiological roles | Pathological roles |
| --- | --- | --- |
| IL-2 | Promotes T cell proliferation and the generation of effector and memory T cells (1)  Maintains function of Treg cells via sustaining Foxp3 expression (2)  Suppresses the differentiation of follicular helper T cells (Tfh) and Th17 cells (3) | Drives cirrhosis-associated immune dysfunction by impairing Tfh cells (4)  Acts as a positive stimulator of autoimmunity (5) |
| TNF-α | Promotes tumor cell necrosis by activating caspase-8 (6)  Promotes the production of inflammatory cytokines by neutrophils, macrophages and dendritic cells (7)  Promotes the release of anti-inflammatory cytokines by regulatory T cells, regulatory B cells and myeloid-derived suppressor cells (7) | Exacerbates inflammatory bowel diseases and colitis associated colon cancer (8)  Promotes autoimmune hepatitis (9) |
| IFN-γ (10, 11) | Promotes macrophages to be more responsive to pro-inflammatory stimuli and resistant to anti-inflammatory stimuli (10)  Promotes T cells polarization toward Th1 and Tfh cells rather than Th2 or Th17 cells (10)  Promotes tumor cell lipid peroxidation and ferroptosis (12) | Promotes supernumerary pathogenic CD4^+^ T cells to infiltrate into central nervous system through inflamed blood-brain barrier in relapsing remitting multiple sclerosis (13)  Inhibits liver regeneration (14)  Participate in the pathogenesis of hyperinflammatory (15) |
| IL-17A | Bolsters host defense against certain pathogens at epithelial and mucosal barriers (16) | Participates in the pathogenesis of psoriasis, ankylosing spondylitis, rheumatoid arthritis, multiple sclerosis, and inflammatory bowel diseases (17)  Drives the progression from non-alcoholic fatty liver to non-alcoholic steatohepatitis (18) |
| PD-1 | Inhibits TCR signaling and induces peripheral tolerance (19)  Inhibits Tfh cells recruitment into the follicle for concentrating these cells toward the germinal center territory and optimizing B cell maturation (20) | Contributes to T cell exhaustion in chronic infection and cancer (21, 22) |
| LAG-3 | Suppresses the activation of CD4^+^ T cells responsive to stable peptide and MHC class II (pMHCII) (23, 24) |  |
| CD69 | Promotes T cells retention in the lymph nodes for full activation (25)  Increases mTOR signaling by upregulating amino acid transport during T cell activation (26) | Inhibits endothelial activation in brain damage following an ischemic stroke (27)  Promotes pathogenic tissue resident memory T cells to infiltrate in inflamed tissues (28) |
| CD103 | Enables immune cells to resident locally by binding to E-cadherin (29) | Promotes intraepithelial γδ T cells to release extracellular granzyme (30) |
| T-bet | Promotes the expression of IFN-γ, CXCR3 in CD4^+^ T cells (31)  Promotes the differentiation of age-associated B cells (32) | |
| EOMES | Promotes the development of IL-10 and IFN-γ co-expressed CD4^+^ T cell (33)  Regulates NK cells development and functions in combination with T-bet (34)  Limits tissue resident memory T cells formation by suppressing Hobit expression (35) | |
| Ki-67 | A hallmark of cell proliferation.  Enables independent chromosome motility and efficient interactions with the mitotic spindle by keeping mitotic chromosomes apart after breakdown of the nuclear envelope (36) | |
| TOX | A central regulator for T cell exhaustion (37) | |

# Supplementary Figures


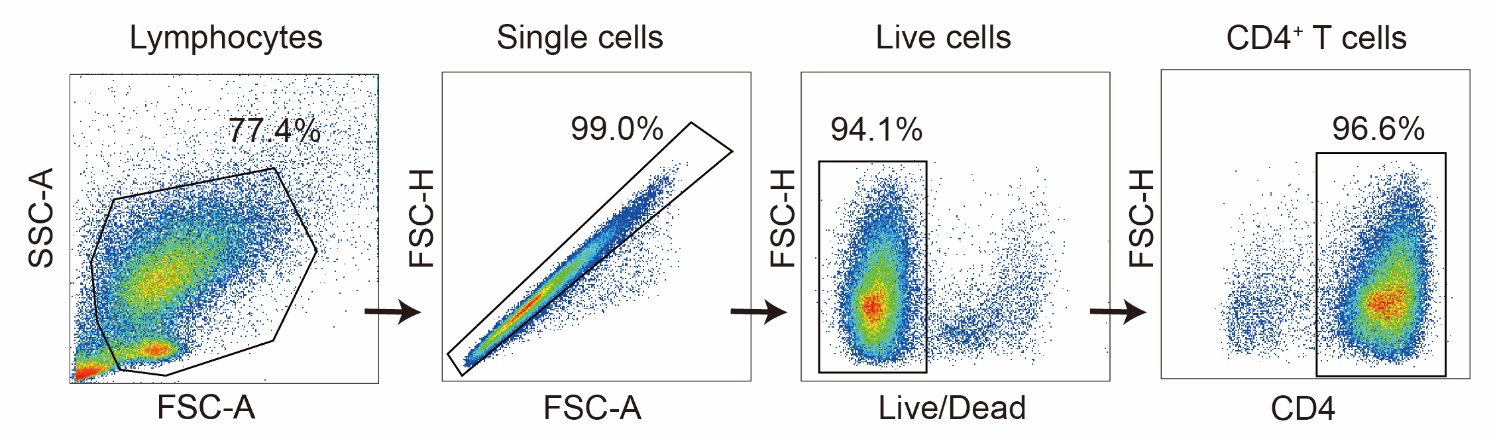


**Figure S1. The purity of isolated CD4^+^ T cells.** The purity of magnetically isolated CD4^+^ T cells was estimated using flow cytometry.

**Reference**

1. Abbas AK, Trotta E, D RS, Marson A, Bluestone JA. Revisiting IL-2: Biology and therapeutic prospects. Sci Immunol. 2018;3(25).

2. Feng Y, Arvey A, Chinen T, van der Veeken J, Gasteiger G, Rudensky AY. Control of the inheritance of regulatory T cell identity by a cis element in the Foxp3 locus. Cell. 2014;158(4):749-63.

3. Pol JG, Caudana P, Paillet J, Piaggio E, Kroemer G. Effects of interleukin-2 in immunostimulation and immunosuppression. J Exp Med. 2020;217(1).

4. Basho K, Zoldan K, Schultheiss M, Bettinger D, Globig AM, Bengsch B, et al. IL-2 contributes to cirrhosis-associated immune dysfunction by impairing follicular T helper cells in advanced cirrhosis. J Hepatol. 2021;74(3):649-60.

5. Sharma R, Fu SM, Ju ST. IL-2: a two-faced master regulator of autoimmunity. J Autoimmun. 2011;36(2):91-7.

6. Aggarwal BB. Signalling pathways of the TNF superfamily: a double-edged sword. Nat Rev Immunol. 2003;3(9):745-56.

7. Salomon BL. Insights into the biology and therapeutic implications of TNF and regulatory T cells. Nat Rev Rheumatol. 2021;17(8):487-504.

8. Jones-Hall YL, Nakatsu CH. The Intersection of TNF, IBD and the Microbiome. Gut Microbes. 2016;7(1):58-62.

9. Bovensiepen CS, Schakat M, Sebode M, Zenouzi R, Hartl J, Peiseler M, et al. TNF-Producing Th1 Cells Are Selectively Expanded in Liver Infiltrates of Patients with Autoimmune Hepatitis. J Immunol. 2019;203(12):3148-56.

10. Ivashkiv LB. IFNgamma: signalling, epigenetics and roles in immunity, metabolism, disease and cancer immunotherapy. Nat Rev Immunol. 2018;18(9):545-58.

11. Hu X, Ivashkiv LB. Cross-regulation of signaling pathways by interferon-gamma: implications for immune responses and autoimmune diseases. Immunity. 2009;31(4):539-50.

12. Wang W, Green M, Choi JE, Gijon M, Kennedy PD, Johnson JK, et al. CD8(+) T cells regulate tumour ferroptosis during cancer immunotherapy. Nature. 2019;569(7755):270-4.

13. Deczkowska A, Baruch K, Schwartz M. Type I/II Interferon Balance in the Regulation of Brain Physiology and Pathology. Trends Immunol. 2016;37(3):181-92.

14. Horras CJ, Lamb CL, Mitchell KA. Regulation of hepatocyte fate by interferon-gamma. Cytokine Growth Factor Rev. 2011;22(1):35-43.

15. De Benedetti F, Prencipe G, Bracaglia C, Marasco E, Grom AA. Targeting interferon-gamma in hyperinflammation: opportunities and challenges. Nat Rev Rheumatol. 2021;17(11):678-91.

16. Jin W, Dong C. IL-17 cytokines in immunity and inflammation. Emerg Microbes Infect. 2013;2(9):e60.

17. Mills KHG. IL-17 and IL-17-producing cells in protection versus pathology. Nat Rev Immunol. 2022.

18. Moreno-Fernandez ME, Giles DA, Oates JR, Chan CC, Damen M, Doll JR, et al. PKM2-dependent metabolic skewing of hepatic Th17 cells regulates pathogenesis of non-alcoholic fatty liver disease. Cell Metab. 2021;33(6):1187-204 e9.

19. Salmaninejad A, Valilou SF, Shabgah AG, Aslani S, Alimardani M, Pasdar A, et al. PD-1/PD-L1 pathway: Basic biology and role in cancer immunotherapy. J Cell Physiol. 2019;234(10):16824-37.

20. Shi J, Hou S, Fang Q, Liu X, Liu X, Qi H. PD-1 Controls Follicular T Helper Cell Positioning and Function. Immunity. 2018;49(2):264-74 e4.

21. Pauken KE, Torchia JA, Chaudhri A, Sharpe AH, Freeman GJ. Emerging concepts in PD-1 checkpoint biology. Semin Immunol. 2021;52:101480.

22. Maruhashi T, Sugiura D, Okazaki IM, Okazaki T. LAG-3: from molecular functions to clinical applications. J Immunother Cancer. 2020;8(2).

23. Maruhashi T, Okazaki IM, Sugiura D, Takahashi S, Maeda TK, Shimizu K, et al. LAG-3 inhibits the activation of CD4(+) T cells that recognize stable pMHCII through its conformation-dependent recognition of pMHCII. Nat Immunol. 2018;19(12):1415-26.

24. Maruhashi T, Sugiura D, Okazaki IM, Shimizu K, Maeda TK, Ikubo J, et al. Binding of LAG-3 to stable peptide-MHC class II limits T cell function and suppresses autoimmunity and anti-cancer immunity. Immunity. 2022;55(5):912-24 e8.

25. Cibrian D, Sanchez-Madrid F. CD69: from activation marker to metabolic gatekeeper. Eur J Immunol. 2017;47(6):946-53.

26. Cibrian D, Saiz ML, de la Fuente H, Sanchez-Diaz R, Moreno-Gonzalo O, Jorge I, et al. CD69 controls the uptake of L-tryptophan through LAT1-CD98 and AhR-dependent secretion of IL-22 in psoriasis. Nat Immunol. 2016;17(8):985-96.

27. Brait VH, Miro-Mur F, Perez-de-Puig I, Notario L, Hurtado B, Pedragosa J, et al. CD69 Plays a Beneficial Role in Ischemic Stroke by Dampening Endothelial Activation. Circ Res. 2019;124(2):279-91.

28. Mueller SN, Mackay LK. Tissue-resident memory T cells: local specialists in immune defence. Nat Rev Immunol. 2016;16(2):79-89.

29. Agace WW, Higgins JM, Sadasivan B, Brenner MB, Parker CM. T-lymphocyte-epithelial-cell interactions: integrin alpha(E)(CD103)beta(7), LEEP-CAM and chemokines. Curr Opin Cell Biol. 2000;12(5):563-8.

30. Hu MD, Golovchenko NB, Burns GL, Nair PM, Kelly TJt, Agos J, et al. gammadelta Intraepithelial Lymphocytes Facilitate Pathological Epithelial Cell Shedding Via CD103-Mediated Granzyme Release. Gastroenterology. 2022;162(3):877-89 e7.

31. Lazarevic V, Glimcher LH. T-bet in disease. Nat Immunol. 2011;12(7):597-606.

32. Johnson JL, Rosenthal RL, Knox JJ, Myles A, Naradikian MS, Madej J, et al. The Transcription Factor T-bet Resolves Memory B Cell Subsets with Distinct Tissue Distributions and Antibody Specificities in Mice and Humans. Immunity. 2020;52(5):842-55 e6.

33. Zhang P, Lee JS, Gartlan KH, Schuster IS, Comerford I, Varelias A, et al. Eomesodermin promotes the development of type 1 regulatory T (TR1) cells. Sci Immunol. 2017;2(10).

34. Simonetta F, Pradier A, Roosnek E. T-bet and Eomesodermin in NK Cell Development, Maturation, and Function. Front Immunol. 2016;7:241.

35. Parga-Vidal L, Behr FM, Kragten NAM, Nota B, Wesselink TH, Kavazovic I, et al. Hobit identifies tissue-resident memory T cell precursors that are regulated by Eomes. Sci Immunol. 2021;6(62).

36. Cuylen S, Blaukopf C, Politi AZ, Muller-Reichert T, Neumann B, Poser I, et al. Ki-67 acts as a biological surfactant to disperse mitotic chromosomes. Nature. 2016;535(7611):308-12.

37. Khan O, Giles JR, McDonald S, Manne S, Ngiow SF, Patel KP, et al. TOX transcriptionally and epigenetically programs CD8(+) T cell exhaustion. Nature. 2019;571(7764):211-8.
